# A new integrated behavioural intervention for knee osteoarthritis: development and pilot study

**DOI:** 10.1186/s12891-021-04389-0

**Published:** 2021-06-08

**Authors:** Stephen J. Preece, Nathan Brookes, Anita E. Williams, Richard K. Jones, Chelsea Starbuck, Anthony Jones, Nicola E. Walsh

**Affiliations:** 1 Centre for Health Sciences Research, University of Salford, Manchester, M6 6PU UK; 2 Physiotherapy Department, Salford Royal NHS Foundation Trust, Salford, M6 8HD UK; 3 Human Pain Research Group, University of Manchester, Clinical Sciences Building, Salford Royal NHS Foundation Trust, Salford, M6 8HD UK; 4 Faculty of Health and Applied Sciences, University of the West of England, Bristol, BS16 1DD UK

**Keywords:** Knee osteoarthritis, Intervention, Behaviour change, Biopsychosocial, Biomechanical, Pain, Co-contraction, EMG

## Abstract

**Background:**

Exercise-based approaches have been a cornerstone of physiotherapy management of knee osteoarthritis for many years. However, clinical effects are considered small to modest and the need for continued adherence identified as a barrier to clinical efficacy. While exercise-based approaches focus on muscle strengthening, biomechanical research has identified that people with knee osteoarthritis over activate their muscles during functional tasks. Therefore, we aimed to create a new behavioural intervention, which integrated psychologically informed practice with biofeedback training to reduce muscle overactivity, and which was suitable for delivery by a physiotherapist.

**Methods:**

Through literature review, we created a framework linking theory from pain science with emerging biomechanical concepts related to overactivity of the knee muscles. Using recognised behaviour change theory, we then mapped a set of intervention components which were iteratively developed through ongoing testing and consultation with patients and physiotherapists.

**Results:**

The underlying framework incorporated ideas related to central sensitisation, motor responses to pain and also focused on the idea that increased knee muscle overactivity could result from postural compensation. Building on these ideas, we created an intervention with five components: making sense of pain, general relaxation, postural deconstruction, responding differently to pain and functional muscle retraining. The intervention incorporated a range of animated instructional videos to communicate concepts related to pain and biomechanical theory and also used EMG biofeedback to facilitate visualization of muscle patterns. User feedback was positive with patients describing the intervention as enabling them to “create a new normal” and to be “in control of their own treatment.” Furthermore, large reductions in pain were observed from 11 patients who received a prototype version of the intervention.

**Conclusion:**

We have created a new intervention for knee osteoarthritis, designed to empower individuals with capability and motivation to change muscle activation patterns and beliefs associated with pain. We refer to this intervention as Cognitive Muscular Therapy. Preliminary feedback and clinical indications are positive, motivating future large-scale trials to understand potential efficacy. It is possible that this new approach could bring about improvements in the pain associated with knee osteoarthritis without the need for continued adherence to muscle strengthening programmes.

**Trial registration:**

ISRCTN51913166 (Registered 24-02-2020, Retrospectively registered).

**Supplementary Information:**

The online version contains supplementary material available at 10.1186/s12891-021-04389-0.

## Background

Knee osteoarthritis (OA) is a chronic long-term condition that results in pain, disability and reduced quality of life [1]. This condition affects a large proportion of individuals, with a global age-standardised prevalence for knee OA estimated to be 3.8% [2]. Indeed, it has been estimated that 10% of the population over the age of 55 will be diagnosed with knee OA [3]. For many patients, conservative treatments do not provide sufficient long-term relief and they choose to undergo total knee replacement. However, as populations age and rates of obesity (a known risk factor [4]) rise, the increasing need for surgical management is putting healthcare systems under considerable strain. Given this huge societal cost, along with the individual burden associated with the disease, there is an urgent need to explore new conservative methods to manage knee OA.

The universally recommended first line of clinical management for knee OA is a physiotherapist-delivered exercise programme. These programmes typically consist of muscle strengthening, advice to remain active [5] along with coping skills [6] and education about self-management. While this approach is supported by large-scale trials [7] and incorporated into national guidelines [8], the magnitude of clinical effect is considered moderate to small [9] and is known to diminish over time [10]. Exercise programmes which consist of two strengthening sessions per week [11], the minimum recommended by the ACSM [12], typically provide a 25–30% reduction in pain and/or function [7]. Furthermore, research has demonstrated that for approximately 40% of patients, exercise-based approaches do not provide any meaningful clinical [13] improvement in symptoms [7]. While adherence has been identified as an issue which may lower the true effectiveness of exercise-based approaches [14], it is unlikely to explain why, for a relatively large number of people, exercise provides no relief from knee OA pain.

While current guidelines focus on the use of exercises to improve strength, there is clear evidence that people with knee OA over activate their muscles during functional tasks [15–17]. This overactivity is characterised by both increased amplitude [18] and prolonged duration [16] of the knee flexor and extensor muscles. Biomechanical studies have investigated the potential effects of muscle overactivity, typically quantifying this phenomenon using a co-contraction index [19]. Increased co-contraction has been linked to increased pain [20], elevated joint load [21], a more rapid rate of cartilage loss [22] and an increase in the likelihood that patients will opt for a knee replacement at 5-year follow up [23]. Given these findings, muscle overactivity is likely to increase the mechanical stress on the articular surface, the bone, joint capsule and periarticular structures and therefore may increase nociceptive input, exacerbating pain [24]. It is therefore important to understand the potential of conservative management techniques which focus on reducing muscle overactivity.

Psychosocial factors have been linked with clinical pain/disability in knee OA. For example, catastrophising [25] and anxiety [26] have been associated with pain intensity and kinesiophobia linked to physical function [27]. Given these links, a number of physiotherapy interventions have been developed which integrate psychological techniques [28, 29] with muscle retraining. This approach is in line with the use of a holistic approach addressing both biomedical and psychosocial factors for the management of chronic low back pain [30, 31]. For example, integrated interventions for knee OA have incorporated a behavioural graded activity programme [32] or have included self-management components to provide reassurance about the value of exercise in OA [11, 33]. However, these interventions have focused primarily on muscle strength training. Therefore, it is unclear whether improved clinical outcomes would be obtained if psychological techniques were integrated with training to reduce muscle overactivity.

This paper describes the development of a new behavioural intervention for knee OA. This intervention integrates psychosocial concepts with emerging biomechanical theory relating to potential drivers of muscle overactivity. The overall aim was to create an intervention that was appropriate for facilitation by a suitably trained physiotherapist and was deliverable within UK NHS resources. In addition to describing the development process and final intervention, we also include some preliminary clinical findings.

## Methods

The structure of the results section follows the guidelines for reporting intervention development studies set out by Duncan et al. [34]. Firstly, we report on the context, purpose, setting and target population (Section 1) after which we provide an overview of how published intervention development approaches contributed to our thinking (Section 2). In Section 3, we describe stakeholder contributions, and, in Section 4, we outline the theoretical ideas which underpin the new intervention. We then outline guiding principles which were prioritised during development (Section 5) and describe in detail the five components of the final intervention (Section 6). Section 7 provides insight into the evolution of the intervention after which we describe potential modifications for subgroups as well as uncertainties (Section 8). At the end of the results section, we present preliminary clinical findings (Section 9) and user perceptions (Section 10).

In order to develop our new intervention, we recruited 21 patients (10 female) with knee OA (mean (SD) age 61 (10) years), who received at least two face-to-face clinical sessions. Of these 21 patients, 11 received five or six sessions of a fully formed version of the intervention. Patients were included if they satisfied the ACR criteria [35] at the time of participation and had experienced knee OA pain for at least 6 months duration. All patients were competent users of the internet. In addition to the patients with knee OA, we recruited 45 healthy individuals in order to create a database of healthy EMG templates. All participants provided informed consent to participate and ethical approval was obtained from a UK NHS research ethics committee (18/NW/0282). All procedures were performed in accordance with the Declaration of Helsinki.

## Results

### Context, purpose, setting and target population

The remit was to create a behaviour change intervention for knee OA which was suitable for delivery by an appropriately trained physiotherapist within a UK NHS outpatient clinic. As the UK NHS is a resource-limited healthcare setting, a total of six face-to-face clinical sessions was considered the maximum feasible. The aim was to create an intervention that would be appropriate for any level of knee OA severity, provided there was no significant impairment in mobility, defined as an inability to walk at least 100 m unaided.

### Overview of the intervention development process

We combined a range of different approaches in developing our new intervention [36]. Following the framework of intervention mapping [37], we followed a process that allowed us to define specific changeable determinants of behaviour that had the potential to exacerbate pain in people with knee OA. This process consisted of a review of biomechanical concepts relating to muscle overactivity, a review of psychosocial theory related to chronic musculoskeletal pain and an exploration of patient beliefs. Our aim was to develop an intervention which was consistent with the COM-B (capability, opportunity and motivation) model [38] which has been recommended for individual-level behaviour change interventions [39]. Throughout intervention development, we adopted a co-design/partnership approach to ensure that the views of patients and physiotherapists were fully represented.

**Fig. 1:**
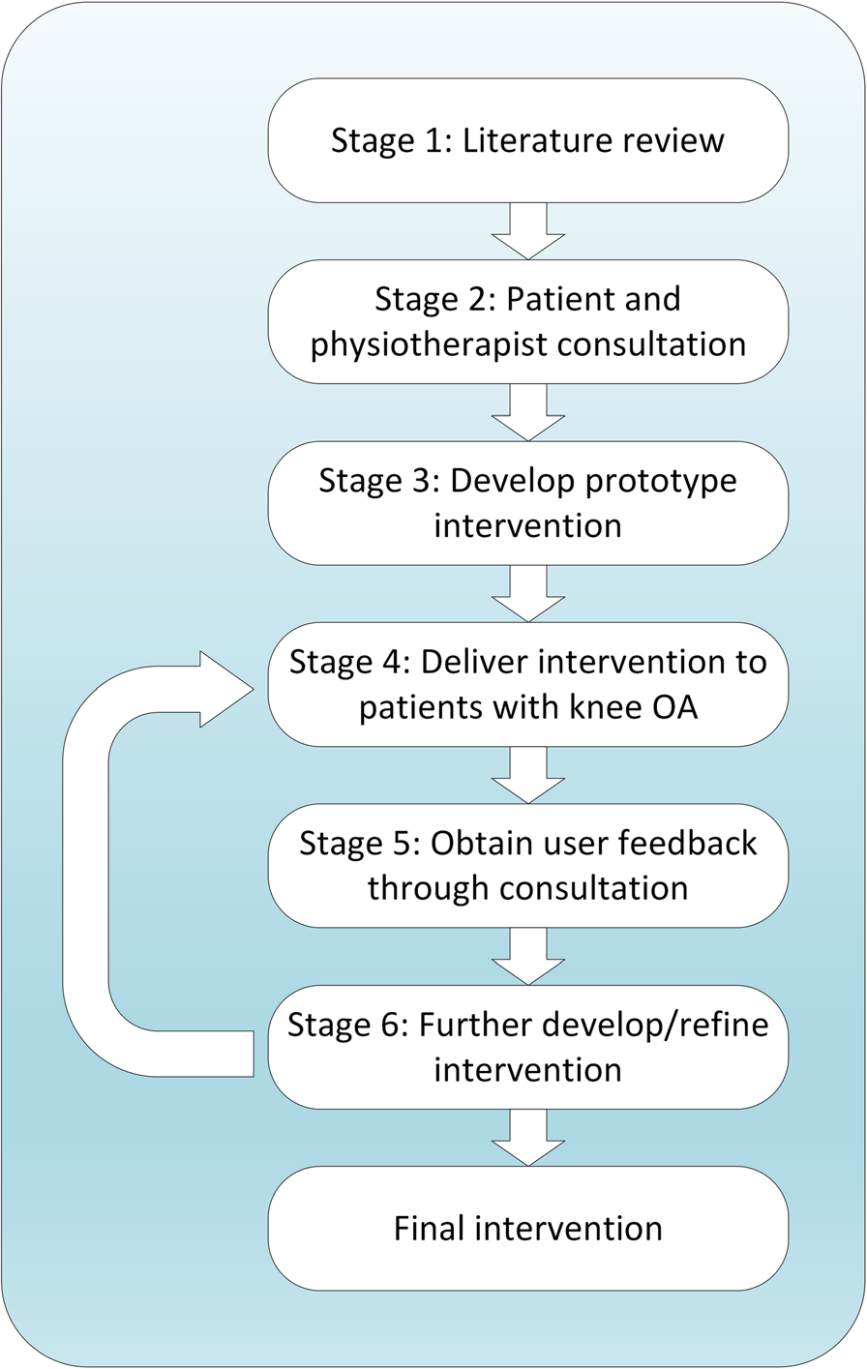
Schematic diagram to show the stages of intervention development

Figure 1 illustrates the stages of intervention development. Following an in-depth literature review (Stage 1), we presented our findings to a group of four patients with knee OA and also a group of four physiotherapists. This consultation (Stage 2) allowed us to explore user perceptions of the theory and to understand beliefs and behaviours which were related to knee OA pain. An initial prototype of the intervention was then created (Stage 3). Between two and six sessions of this prototype intervention were delivered to 10 patients with knee OA by the lead physiotherapist (NB) (Stage 4). User feedback on this initial prototype (Stage 5) was obtained via three mechanisms: feedback directly to the physiotherapist after each session; interviewing of patients by a qualitative researcher; and through co-design workshops involving both physiotherapists and patients (see section below).

In order to respond to user feedback, the intervention was again refined/developed (Stage 6). This second iteration of the intervention was delivered to a further six patients (5–6 sessions), again by the lead physiotherapist (Stage 4). Following this delivery, we used the same three mechanisms to obtain user feedback (Stage 5), again refining the intervention as appropriate. At the end of this second iteration, the intervention was delivered to a further five patients (5–6 sessions). During this final period of testing, only minor refinements were made in response to feedback made directly to the physiotherapist.

### Stakeholders contribution to intervention development

Through our initial user consultation (Stage 2, Fig. 1), we explored patient’s perceptions of their knee condition. This exploration was carried out following a presentation of the theory, allowing patients to contextualise their own experiences and to reflect on possible explanations for pain that were hitherto unknown to them. Through consultation with physiotherapists, we were able to understand potential barriers and facilitators for delivery within the UK NHS, for example, the need to create an intervention which could be delivered through six clinical sessions. Discussions were analysed using a framework developed to understand the acceptability of healthcare interventions [40] and the findings used to specify changeable determinants of behaviour. The outputs from these discussions were also used to inform the guiding principles which were prioritised during intervention development. Through this process, we created a specification for the initial intervention prototype.

At the end of each physiotherapy session, we recorded the patient’s view of the different aspects of the intervention, such as educational materials, and whether there has been any change in their pain-related beliefs. In addition, a subset of three patients were interviewed by a qualitative researcher (NW or AW) to gain further insight into user perspectives and potential health benefits. With both these approaches, thematic analysis [41] was used to specify how the intervention could be improved. Three co-design workshops were held during intervention development (Stage 5, Fig. 1) involving at least four physiotherapists and at least four patients. Following a presentation of the theory and demonstration of the intervention, we ran separate and combined focus groups (with patients/physiotherapists) to understand user perspectives.

We consulted with a patient advisory committee on various aspects of intervention development and research design. This group consisted of four individuals with a history (>5 y) of knee OA. The group provided input on aspects such as the format of the co-design workshops, participant information resources and specifications for subsequent iterations of the intervention. No PPI members were included in the final 11 participants who received a fully formed version of the intervention and for whom we report clinical outcomes.

### Theoretical components and patient beliefs

The theoretical framework for the intervention was created from three separate components. These components were postural mechanisms which could underlie muscle overactivity, motor responses to pain and altered central pain processing. As the aim was to create a completely new intervention, we drew on emerging evidence and theory, ideas from other chronic musculoskeletal disease, e.g. low back pain, and also incorporated the findings of ongoing biomechanics research in our lab.

#### Muscle overactivity through postural mechanisms

**Fig. 2:**
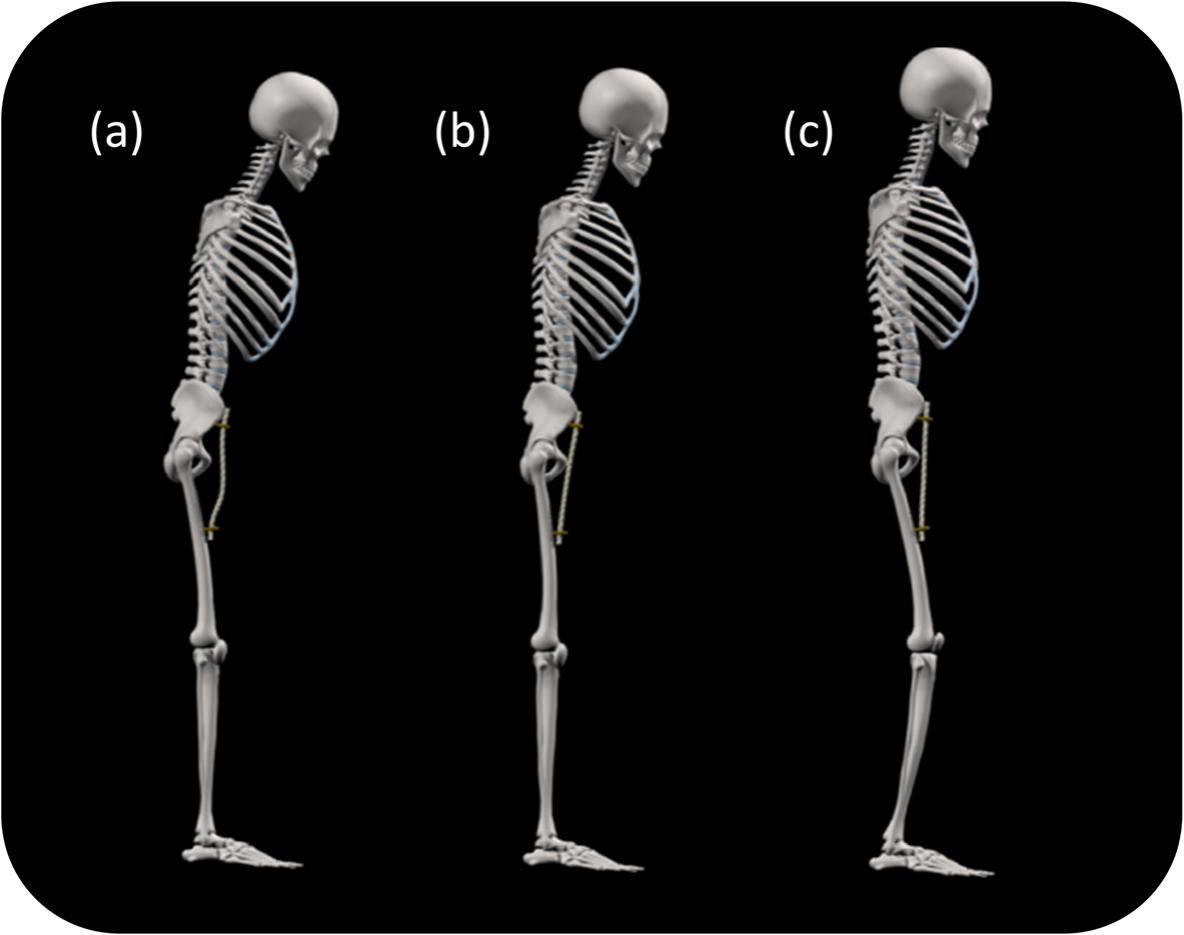
(**a, b**) A passively stiff hip flexor (illustrated as a rope) prevents the pelvis returning to a neutral position in standing. (**c**) Biomechanical compensation for a passively stiff hip flexor, consisting of a flexed hip, knee and ankle and an increased lumbar lordosis. Note there is still a slight flexion of the trunk. A full animation of this pattern can be viewed at: www.cogmustherapy.com/BMC_example_1

There is clear evidence of altered postural alignment in people with knee OA. This is characterised by a flexed posture [42], altered lumbo-pelvic alignment [43] and an increase in forward spinal inclination [44, 45]. Given the potential link between intersegmental muscle length and posture [46, 47], these findings may indicate that people with knee OA have some form of muscle imbalance of the hip/trunk muscles. This idea is consistent with research showing that people with knee OA have increased passive stiffness of the hip flexor muscles [48]. Such increased stiffness will limit posterior pelvic rotation (pelvic tilt) [49], preventing the pelvis from returning to a neutral position in upright standing (Fig. 2a-b). Without any biomechanical compensation, a passively stiff hip flexor will increase forward spinal inclination, shifting the centre of mass anteriorly (Fig. 2b). Increased hip extensor (e.g. hamstring) activity will then be required to maintain upright standing. However, we suggest that such an extremely flexed position is unlikely to be adopted. Instead, it is likely that an individual with a passively stiff hip flexor will “biomechanically compensate”, by flexing the hip, knee and ankle [42] and by increasing lumbar lordosis in an attempt to stand upright and maintain gaze alignment (Fig. 2c). This compensation will require an increase in quadriceps muscle activity to maintain a flexed knee in standing.

**Fig. 3:**
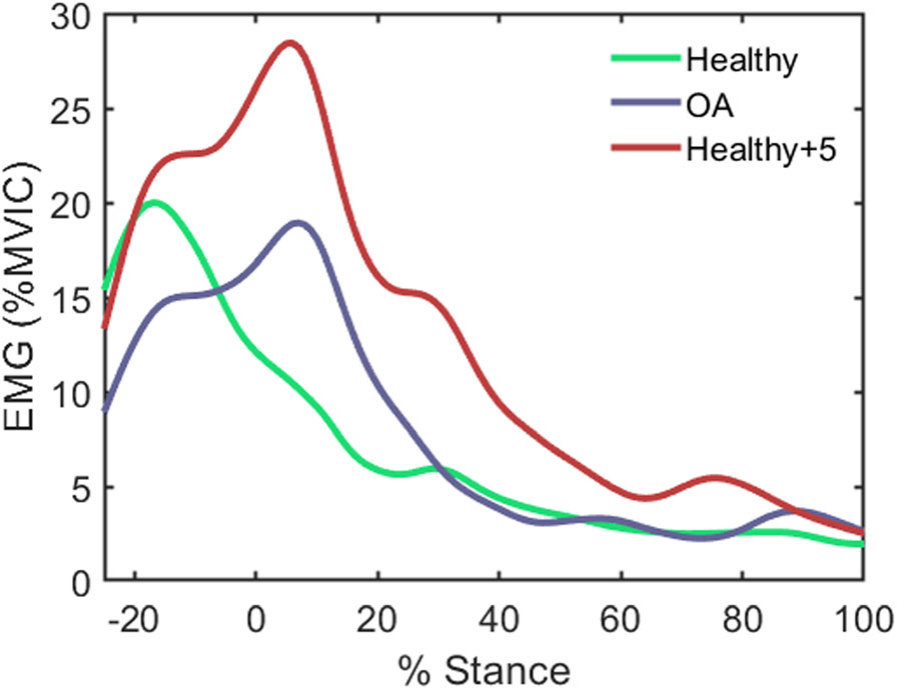
Medial hamstring EMG during walking in people with knee OA (blue), in healthy people (green) and in healthy people after instruction to increase trunk flexion by 5° (red). Note how the muscle pattern in the healthy people changes dramatically, becoming similar to the OA pattern, with increased trunk flexion. MVIC refers to maximal voluntary isometric contraction

As well as influencing muscle activity in standing, postural mechanisms may also underlie, to some degree, muscle overactivity during walking, which is exhibited by people with knee OA [50]. Emerging research from our lab supports this idea, showing a link between trunk flexion in walking and passive stiffness of the hip flexor muscles [48] and higher knee muscle activity in healthy people who walk with more trunk inclination [51]. Importantly, we have observed that people with knee OA walk with an increased flexion (forward lean) of the trunk [52]. Critically, when we instruct healthy people to increase their trunk flexion by only 5°, knee flexor muscle patterns become similar to those associated with knee OA [50, 53] (Fig. 3).

**Fig. 4:**
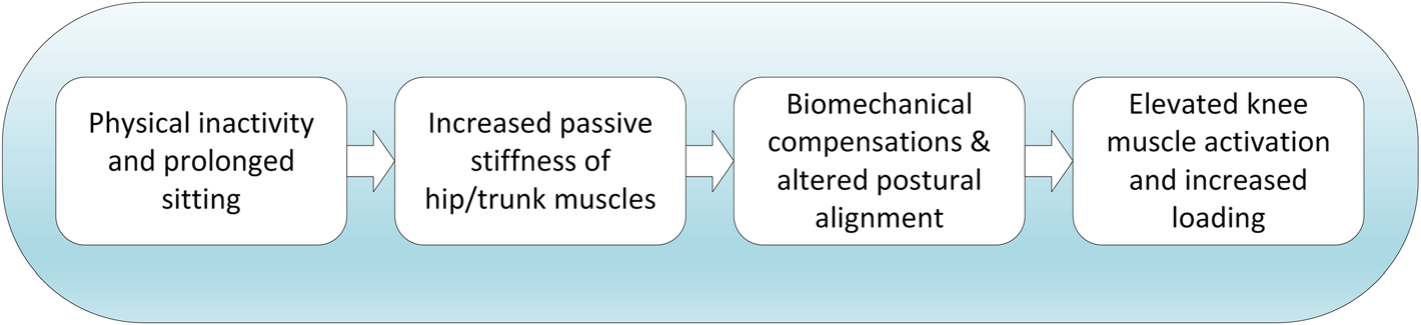
Postural framework to explain elevated knee muscle activation from increased passive stiffness of hip/trunk muscles

Central to the postural mechanisms outline above is the idea that overactivity of the knee muscles could result from increased passive stiffness of hip/trunk muscles. It has been suggested that chronic understretch [54, 55] can lead to increased passive stiffness of muscles. This is consistent with our research showing limited passive hip extension in healthy people who sit for prolonged periods and who are physically inactive [56]. It may also explain the observation of reduced transverse plane motion of the thoracic spine which would result from increased stiffness of the abdominal muscles [57]. Figure 2 depicts how passive stiffness of hip flexor muscles could trigger compensatory changes in knee muscle activity. Similarly, increased stiffness of the abdominal muscles will reduce the capacity of the rib cage to move superiorly, relative to the pelvis [58], and could therefore affect postural alignment in standing, potentially triggering knee muscle overactivity. Given these ideas, we integrated the idea of a link between sedentary behaviour, increased passive stiffness of hip/trunk muscles, biomechanical compensation and knee muscle overactivity (Fig. 4).

#### Central modulation of the pain experience

There is strong evidence to support the idea that people with knee OA are oversensitive to pain in general [59, 60]. This so called central sensitisation [61] can occur through a range of mechanisms, such as amplification of afferent nociceptive impulses from peripheral receptors or alteration of sensory processing in the brain. While intense and continued nociceptive input is known to cause central sensitisation [62], it is also possible that emotional responses to pain [63] or pain expectations can influence sensitisation. This idea is consistent with research which has demonstrated that psychosocial factors can mediate the association between hyperalgesia and knee pain [64]. Characteristics such as pain catastrophising [65], kinesiophobia (fear of movement) [25], helplessness [66], reduced self-efficacy [67], anxiety [26] and depression [26] have all been shown to be associated with knee OA pain and are likely to play a role in shaping the pain experience. Therefore, we integrated the idea of a relationship between central modulation, the pain experience and emotional responses to pain.

#### Motor adaptation to pain

Adaptation of the motor system to pain [68] or anticipated pain [69] is a well-recognised phenomenon [70]. Given the consistent observation of longer duration and increased amplitude of EMG in people with knee OA across different tasks [16, 18], it would appear that motor adaptation in this disease is characterised by an overactivity of the knee muscles. Although this strategy may enhance joint stability following acute injury [71], it will increase joint loading [72] and is likely to increase nociceptor input, exacerbating pain, if maintained in the long-term. There is evidence that muscle overactivity in low back pain is related to pain-related fear [73] and pain catastrophising [74]. This indicates that emotional responses and expectations are likely to shape long-term motor adaptation to musculoskeletal pain. While this relationship has not been explored in knee OA, a recent study demonstrated a link between central sensitisation to pain and muscle overactivity [75] in people with this disease. This may suggest a link between motor adaption and central modulation, which as explained above, is likely to be mediated by emotional responses to pain. Given this likely interdependence, we integrated the idea of a relationship between motor adaption, the pain experience, central modulation and emotional responses to pain.

#### The integrated framework

**Fig. 5:**
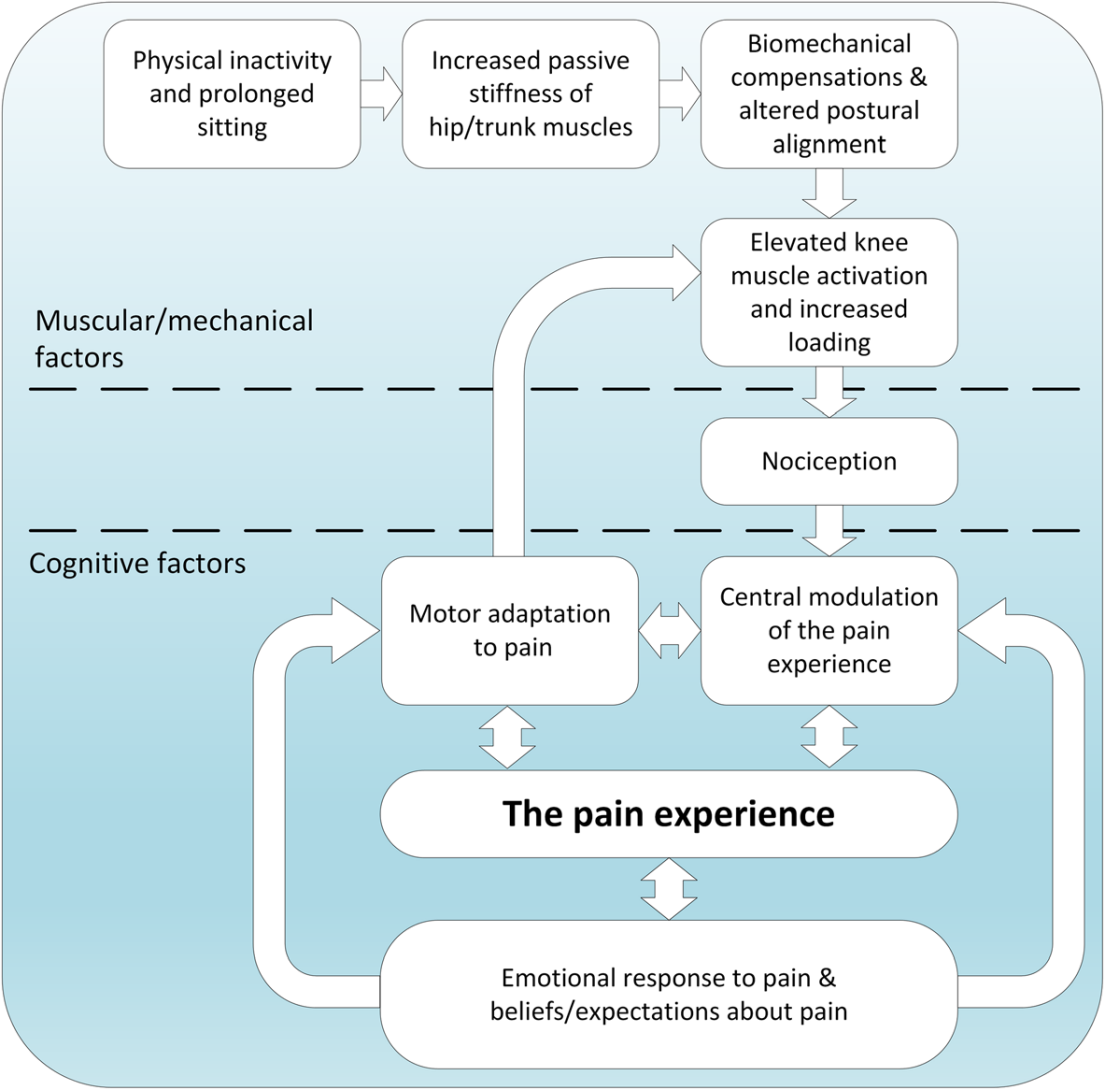
Integrated behavioural framework

Figure 5 shows the fully integrated behavioural framework, obtained by combining the different mechanisms described above. This framework can be divided into muscular/mechanical factors which stimulate nociceptive input and cognitive factors which shape responses to pain and the pain experience. It is important to stress that our proposed framework is not a comprehensive model to explain the onset of knee OA. Clearly, OA pain may have many origins, such as ligament rupture or other traumatic injury [76, 77]. Instead, we have attempted to include factors which could exacerbate knee pain, may relate to patient beliefs, and that could be targeted through an effective behaviour change intervention.

#### Exploration of patient beliefs

Following theoretical development, we consulted with patients (Stage 2, Fig. 1), encouraging them to contextualise their own pain experiences. A range of beliefs were identified which were subsequently confirmed during intervention testing (Stage 5, Fig. 1). Table 1 lists the three beliefs which were universally held amongst patients. In addition to these three beliefs, we identified beliefs relating to the degree in which patients were fearful or anxious about their pain. These beliefs were often markedly different between individuals and this highlighted the need for our final intervention to be individually tailored based on a patient's need. By combining our understanding of patient beliefs with the theoretical framework (Fig. 5), we developed a set of changeable determinants of behaviour. Before we describe this development, we highlight guiding principles for intervention development which were established as part of our consultation.

**Table 1:** Commonly held patient beliefs about knee OA pain

	Pain-related belief
1	My knee OA pain is the inevitable result of wear and tear on the joint and is part of getting old
2	Certain activities (e.g. going downstairs) cause high levels of pain and so I need to avoid them wherever possible
3	Knee OA is a chronic condition and so the pain is something I am always going to have to live with and may get worse in the future

### Guiding principles prioritised during intervention development

To facilitate patient learning, we used digital technologies where possible, working with a local animation studio to create a range of instructional videos. These videos were used to convey intervention theory and to guide practice outside clinical sessions. Each participant was provided with a subset of videos which was tailored (by the physiotherapist) to their individual needs. The format of each clip was the same, being approximately 1 min in length and finishing with a question that reflected the learning outcome, e.g. “Do you understand that increased knee muscle tension could make your knee pain worse?” Participants watched the videos on a tablet computer and had the option of repeating each clip if they did not fully grasp the learning outcome. To facilitate motor relearning, we used EMG biofeedback [78] from the knee muscles. To optimise usability, we created our own software which could be used to visualise simple on-off activity or, alternatively, used to contrast an individual’s muscle pattern with a healthy average EMG profile for a given functional task.

One of our principal aims was to create a behaviour change intervention which would not require longer-term adherence to a prescribed programme of exercise. Rather than conditioning the knee muscles, our intervention was designed to change beliefs about pain and to change muscle patterns, providing patients with capability, opportunity and motivation to self-manage their condition. In this context, the physiotherapist’s role was that of an educator, guiding patients through a tailored, incremental learning process. While patients were required to practice certain procedures in the short-term to facilitate this learning, the ultimate aim was for patients to be able to integrate this learning into daily activity without the need to set aside specific time to practice. A relatively rapid transition from self-directed practice into daily activity was felt to be critical given that exercise adherence has been identified as a major barrier in the physiotherapy management of knee OA [14, 33].

### Intervention components

In response to the integrated theoretical framework (Fig. 5) and the set of pain-related beliefs (Table 1), we mapped a set of five changeable determinants of behaviour (Table 2). Using the taxonomy of behaviour change methods [79], we then identified behaviour change techniques which were appropriate for each determinant (Table 3) and which were incorporated into our five intervention components. These components were: making sense of pain, general relaxation, postural deconstruction, responding differently to pain and functional muscle retraining. Each intervention component was associated with several determinants of behaviour change, corresponding techniques (Table 2) and was specifically tailored to the individual patient. An extensive description of the five intervention components is provided in Additional file 1, with a summary below.

**Table 2:** Changeable determinants of behaviour, behaviour change methods and corresponding intervention components. Each determinant has been mapped back to the COM-B model of behaviour change. (COM-B refers to capability, opportunity and motivation)

Changeable determinant of behaviour	Behaviour change technique	Intervention component	COM-B
Recognise that increased knee muscle activation will increase load on the joint, potentially exacerbating pain.	Persuasive communication	Making sense of pain	Motivation to engage in re-learning of muscle patterns
	Using imagery		
Recognise that emotional factors can impact on central sensitisation and affect the pain experience.	Persuasive communication	Making sense of pain	Motivation and opportunity to challenge pain-related beliefs
	Using imagery Consciousness raising		
Develop awareness of acute muscular response to pain (e.g. knee bracing) and be able to consciously influence these patterns.	Consciousness raising	Making sense of pain	Capability and opportunity to change muscular responses to pain
	Counterconditioning	General relaxation	
	Bio (Feedback)	Responding differently to pain	
Understand the concept of biomechanical compensation and be able to reorganise postural muscle activity in order to minimise knee muscle activation in standing.	Using imagery	General relaxation	Capability and opportunity to change muscular control of posture in standing
	Consciousness raising	Postural deconstruction	
	(Bio)Feedback		
Develop the ability to reduce muscular overactivity during functional tasks, such as walking.	Using imagery	Responding differently to pain	Capability and opportunity to change muscular coordination in everyday tasks
	Consciousness raising		
		Functional muscle retraining	
	Counterconditioning		
	Bio (Feedback)

**Table 3:** The five primary behaviour change methods

Behaviour change method	Definition
Persuasive communication	Guiding individuals toward the adoption of an idea, attitude, or action by using arguments or other means.
Using imagery	Presenting information in a pictorial format will aid the communication of conceptual ideas and facilitate the learning of new motor patterns
Consciousness raising	Providing information and feedback about the causes, consequences, and alternatives for a problem behaviour.
Counter conditioning	Encouraging the learning of healthier behaviours that can substitute for problem behaviours.
Feedback	Giving information to individuals regarding the extent to which they are accomplishing learning or performance.

#### Component 1: making sense of pain

We used persuasive communication and imagery to challenge the erroneous belief that knee OA pain is the inevitable result of “wear and tear”. We then conveyed the idea that increased muscle activation will increase knee loads, potentially increasing pain and that “tensing muscles in response to pain” may also exacerbate pain. We explained that brain processing and psychosocial factors may shape the pain experience (Fig. 5) [80], stressing the need to raise consciousness of both muscular and emotional responses to pain. An example animation can be viewed at www.cogmustherapy.com/BMC_example_2.

#### Component 2: general relaxation

We targeted three easy-to-observe characteristics of relaxation in order to raise consciousness of muscular responses to pain. These were active contraction of the quadriceps muscles, resistance to passive limb movement and low level contraction of the abdominal muscles which can impair diaphragmatic breathing [81]. Using clinical instruction, supplemented with animated videos, patients were encouraged to develop an awareness of low-level patterns of muscular holding, first in lying/sitting and then in standing. EMG biofeedback was used to teach awareness of quadriceps contraction and simple observations of abdominal movement used to guide breathing awareness. An example video can be viewed at www.cogmustherapy.com/BMC_example_3.

#### Component 3: postural deconstruction

In Section 4.1 we outlined mechanisms to explain overactivity of the knee muscles as a compensation for elevated passive stiffness of the hip/trunk muscles (Fig. 4). Given this link, this intervention component specifically addressed patterns of low level postural muscle activity, known as postural tone [82]. This was achieved through a set of clinical procedures which allowed the physiotherapist to unpick (deconstruct) patterns of compensatory tone. These procedures were designed to raise consciousness of hip/trunk muscle stiffness and compensatory tone, and incorporated EMG biofeedback along with instructional animations to communicate biomechanical concepts. Through this process, patients were provided with experiential learning of how to stand with less compensatory knee muscle activity. Given the potential link between physical inactivity and increased passive stiffness of hip/trunk muscles, patients were encouraged to take regular walking exercise and break up periods of prolonged sitting. The physiotherapist also challenged beliefs relating to exercise avoidance.

#### Component 4: responding differently to pain

This intervention component used EMG biofeedback to raise consciousness of inappropriate contraction of the knee muscles which was triggered by pain expectations. Using biofeedback, the patient was taught to down regulate (counter condition) anticipatory muscular contraction, which occurred before initiation of functional movement, e.g. before stepping down. Such muscle patterns are likely to be connected to past experience and beliefs about pain. Therefore, the clinician used this opportunity to continue to explore patient’s beliefs around the causes of pain and encouraged individuals to reflect on their own emotional responses to anticipated pain.

#### Component 5: functional muscle retraining

We created software which facilitated the visualisation of a patient’s EMG profile against a healthy template for different functional tasks. Using this software, the clinician identified periods of inappropriate muscle activity and then used motor imagery [83] to encourage downregulation of knee muscle activity. For example, many people with knee OA exhibit prolonged quadriceps activity into midstance of walking [84]. By using an instruction (for example “imagine a rope pulling the leg forwards as you walk”), the patient learned to associate the specific motor command with the healthy template, receiving continuous EMG biofeedback to guide learning. By working through a range of functional tasks, the clinician challenged beliefs that certain movements should be avoided, providing experiential learning that these tasks could be performed with less muscle activation.

#### Intervention schedule

The final intervention was delivered as a course of six one-to-one clinical sessions (one every two weeks), each lasting 45–60 min and which was augmented with specific tasks that patients completed outside of contact sessions. The first clinical session typically covered making sense of pain (component 1) and general relaxation (component 2). In sessions 2–4, this material was revised, and the patient taught postural deconstruction (component 3) and responding differently to pain (component 4). In the final two sessions, there was more focus on functional muscle retraining (component 5), however, this was determined on individual needs. Outside clinical sessions, patients practised relaxation, postural deconstruction and the use of specific motor commands to influence muscle patterns. They were also encouraged to take regular exercise and to notice their emotional and muscular responses to pain. The ultimate aim of the intervention was to create capability, opportunity and motivation to change behaviour related to knee pain. In line with this philosophy, patients were instructed to gradually integrate as many of the ideas and practices into their activities of daily living, removing the need to dedicate specific time each day to practice.

### The evolution of the intervention

During the two-year development process, we changed the way the intervention was delineated into different components. The initial prototype contained components aligned with specific aspects of the technical development work, such as instructional animations and biofeedback software. However, we later delineated the intervention into components that aligned with learning objectives, such as making sense of pain, general relaxation and functional muscle retraining. We also moved from the original concept of an introductory video to explain psychological and biomechanical concepts, to the practice of producing several short clips (< 1 min). As the intervention progressed, the physiotherapist was able to add these clips to a playlist on a tablet computer (provided to the patient), gradually increasing the information that patients were required to digest after each session.

A major part of the development work was focused on the clinical procedures which formed the basis of the postural deconstruction component of the intervention. After experimenting with numerous strategies, we found that the idea of a “tension point” to be the most effective way to link our conceptual framework with a patient’s kinaesthetic understanding. In line with the focus on postural tone, this idea shifted the objective from that of achieving a distinct postural alignment to a focus on the muscles used to stand erect. Another part of the developmental work focused on the functional muscle retraining. While our original idea had been to guide patients through a set of incremental activities for each task, it proved difficult to break down complex functional movements, such as walking. Therefore, following the preliminary balance training, the use of guided imagery was found to be the most appropriate method for changing motor patterns.

### Intervention modification for subgroups and potential uncertainties

Given that our intervention is tailored to an individual’s needs, we are confident that it would be appropriate for most patients with knee OA. However, we acknowledge that further development would be required for people who are unable to stand unaided or need to use a walking aid. Our intervention was specifically designed for a UK NHS setting and therefore we did not investigate the potential of adapting the number of intervention sessions on an individual basis. However, we suggest that increasing the number of clinical sessions might improve outcomes. Another potential uncertainty is the training a physiotherapist would require in order to deliver this new intervention. Wherever possible, we created supplementary material to facilitate patient learning, used established psychosocial techniques for the management of chronic musculoskeletal pain and used existing clinical physiotherapy assessment methods. Therefore, we suggest that a relatively short training course should prove sufficient and we are currently exploring how to design such a course.

### Preliminary clinical results

The final 11 participants received five or six sessions of the intervention which, although not finalised, was considered sufficiently formed for clinical delivery. All sessions were delivered by the lead physiotherapist (NB). For this group of 11 participants (six male), the mean (SD) age was 60 (9) years, weight 83.7 (18.2) kg and height 1.72 (0.08) m. All satisfied ACR criteria and had a previously confirmed radiographically diagnosis of knee OA (KL grade unavailable). KOOS data were collected from each participant at baseline, 12 week follow up (immediately after the final intervention session) and at a long-term follow up (between 9 and 15 months from baseline). In addition to KOOS pain, we calculated the corresponding WOMAC pain from the final 5 items of the KOOS pain scale in order to facilitate comparison with other studies.

There were large changes in both pain and function immediately following the intervention (Table 4), with a reduction of 55% in KOOS pain, 68% in KOOS function and 69% in WOMAC pain. All participants reported an improvement in WOMAC/KOOS pain above the minimum threshold of 15% [13], with individual improvements in KOOS pain ranging from 33 to 88%. While reductions in average pain appeared to be maintained at long-term follow up, the improvement in KOOS function declined. However, while the 11 participants received five-six intervention sessions, the intervention had not been finalised and it is therefore possible that these data may provide an indication of the minimum effect.

**Table 4:** Mean (SD) change in pain and function across 11 patients

	Baseline measure	12 week follow up			9–15 month follow up		
		Follow up	Change from baseline	% change	Follow up	Change from baseline	% change
KOOS pain	47 (14)	21 (11)	26 (20)	55 (17)	25 (21)	22 (18)	47 (41)
KOOS function	36 (20)	10 (7)	26 (18)	68 (23)	21 (19)	15 (22)	35 (50)
WOMAC pain	8.2 (2.4)	2.5 (2.1)	5.6 (2.2)	69 (20)	4.0 (4.1)	4.2 (3.7)	53 (41)

WOMAC pain data are scored 0–20, with higher scores indicating more pain. KOOS data are scored 0–100 with higher scores indicating more pain/lower function. Note that, for ease of comparison with WOMAC, the KOOS scores have not been transformed

### Patient perceptions of the intervention

Feedback from patients, captured during the final co-design workshop, was universally positive. Patients reported that the intervention allowed them to understand and challenge the way they move and react to pain. They also described the process as allowing them to “create a new normal”, to be “in control of their own treatment” and to feel like they were addressing the “cause not the symptoms.” Patients also commented positively on the “holistic approach” and explained that this put the “patient at the centre rather than the health professional or the treatment”. One participant described the intervention as “genuinely life changing” as it had had both a psychological and physical impact and had resulted in her feeling “more energised.”

Following interviews with three participants, who were within the final 11 patients receiving the fully formed intervention, a number of themes were identified using thematic analysis [41]. Firstly, that the intervention had “changed mind and body”, giving them a new level of conscious awareness of their body movements. Secondly, that “understanding is the key” and that the use of animated videos and EMG biofeedback was invaluable. When combined with individual discussion, this enabled patients to “reset expectations” about their knee pain and give them a new feeling of “responsibility” for their condition. A third theme related to the need to “keep going with the new me”, recognising the importance of continued awareness in daily life. Finally, the importance of an empathic and positive attitude of the therapist was recognised as being crucial in changing patient’s beliefs about pain and guiding them through the learning process.

## Discussion

Our intervention is novel because of the integration of psychological techniques with muscle biofeedback training, specifically designed to target muscle overactivity. Given this integration, we propose a label for the intervention of “Cognitive Muscular Therapy.” EMG biofeedback techniques have been used extensively in rehabilitation [85]. However, our approach is especially unique because of the use of biofeedback to raise consciousness of muscle overactivity related to pain expectations (component 4) and the use of postural deconstruction to reduce muscle overactivity in standing (component 3). We acknowledge that some of the biomechanical underpinnings for the intervention are based on emerging concepts rather than on unequivocal evidence. Nevertheless, our preliminary clinical findings and positive patient feedback motivate further research to understand the links between muscle overactivity, motor adaption to pain and central sensitisation.

**Fig. 6:**
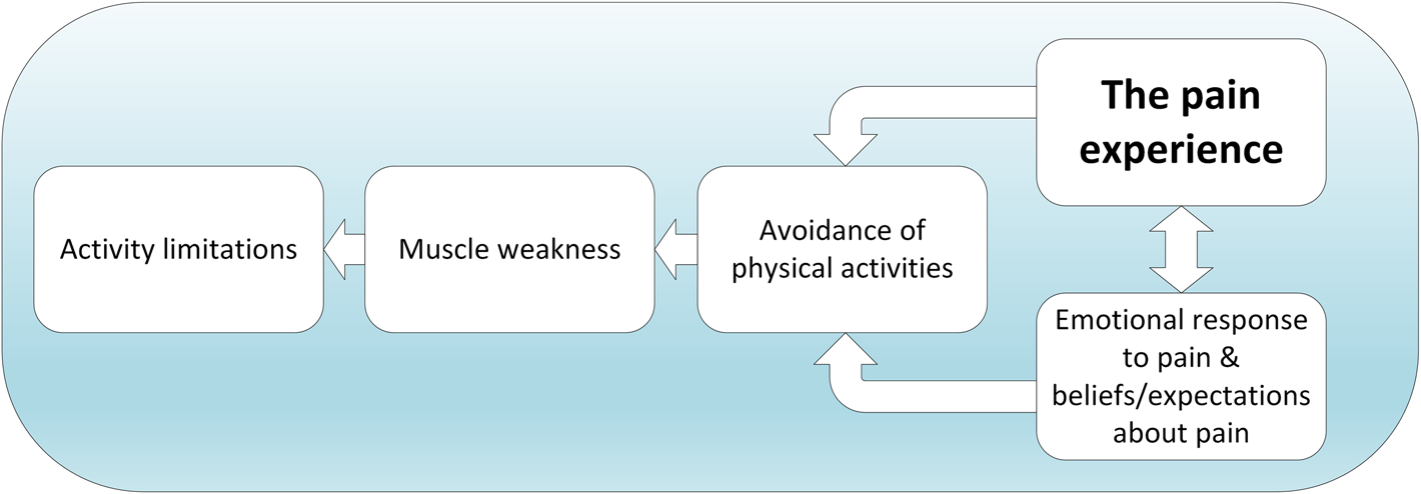
The avoidance model in knee osteoarthritis (adapted from [87])

People with knee OA are known to exhibit weakness of the quadriceps muscles [86]. There is strong evidence to support the idea that such reductions in strength result directly from activity avoidance [87]. With the so-called avoidance model, the patient with knee OA experiences pain during activities, leading to the expectation that further activity will cause pain and the subsequent avoidance of activities (Fig. 6). This model is consistent with the theoretical framework we used to develop our intervention (Fig. 5). However, rather than directly target muscle weakness, our approach was to challenge beliefs and to provide experiential learning that daily activities could be performed with less muscle overactivity. It is therefore likely that clinical improvements from Cognitive Muscular Therapy occur through different mechanisms to those obtained via muscle strengthening [88]. Nevertheless, it is possible that by encouraging patients to resume normal physical activity, there could be long-term improvements in strength. Clearly, further testing is required to explore this idea.

Other emerging treatment paradigms for knee OA focus on gait retraining through the use of simple instruction to change foot progression angle, alter step width or medialise the knee position [89]. However, while these approaches have been shown to reduce the load on the medial compartment [89], they are associated with muscle overactivity [90]. In contrast, Cognitive Muscular Therapy was specifically designed to reduce elevated muscle activity. It is possible that this treatment target may lead to a reduction in focal bone loading, which has been linked to pain in knee OA [91]. However, further research is required to explore this idea.

We acknowledge that our intervention did not specifically incorporate the full range of social and lifestyle factors that may shape the pain experience. For example, factors such as obesity [92], sleep disturbance [93] and possible stressful life situations [94] have been linked to chronic pain. Delineation of these boundaries was deemed appropriate to create an intervention which could be delivered within six face-to-face sessions. Nevertheless, we are confident that it would be straightforward to augment our intervention with other approaches for addressing general health, social and lifestyle factors, such as weight management, cognitive behavioural therapy and more extensive social support. However, we would emphasise that our intervention is designed to teach patients how to manage and react to pain differently and may therefore facilitate self-management of co-presenting musculoskeletal pain [95].

## Conclusion

We have created a completely new behavioural intervention for knee OA which integrates ideas from pain science, biomechanics and health psychology, and which can be delivered by a physiotherapist. We propose to refer to this intervention as Cognitive Muscular Therapy. The intervention contains a focus on changing muscle patterns and teaching patients about how their beliefs and behaviours can shape the pain experience. The intervention is consistent with the COM-B model of behaviour change. User feedback was incredibly positive. However, while encouraging, our preliminary clinical data does not constitute proof of effectiveness. Therefore, larger trials are now required to understand whether this intervention could bring about long-term improvements in the pain associated with knee OA when delivered either within the UK NHS or other healthcare settings.

## Supplementary Information

**Additional file 1.** Full description of the final intervention.

## Data Availability

Full KOOS data collected from the final 11 participants can be downloaded at the following link: https://doi.org/10.17866/rd.salford.14709276.

## References

[1] Woolf AD, Pfleger B (2003). Burden of major musculoskeletal conditions. Bull World Health Organ.

[2] Cross M, Smith E, Hoy D, Nolte S, Ackerman I, Fransen M, Bridgett L, Williams S, Guillemin F, Hill CL, Laslett LL, Jones G, Cicuttini F, Osborne R, Vos T, Buchbinder R, Woolf A, March L (2014). The global burden of hip and knee osteoarthritis: estimates from the global burden of disease 2010 study. Ann Rheum Dis.

[3] Peat G, McCarney R, Croft P (2001). Knee pain and osteoarthritis in older adults: a review of community burden and current use of primary health care. Ann Rheum Dis.

[4] King LK, March L, Anandacoomarasamy A (2013). Obesity & osteoarthritis. Indian J Med Res.

[5] Osteoarthritis: care and management (2014). Clinical guideline [CG177]. National Institute for Clinical Excellence. 31869054

[6] Bennell KL, Ahamed Y, Jull G, Bryant C, Hunt MA, Forbes AB, Kasza J, Akram M, Metcalf B, Harris A, Egerton T, Kenardy JA, Nicholas MK, Keefe FJ (2016). Physical therapist-delivered pain coping skills training and exercise for knee osteoarthritis: randomized controlled trial. Arthritis Care Res.

[7] Hurley MV, Walsh NE, Mitchell H, Nicholas J, Patel A (2012). Long-term outcomes and costs of an integrated rehabilitation program for chronic knee pain: a pragmatic, cluster randomized, controlled trial. Arthritis Care Res.

[8] Osteoarthritis: care and management [CG177]: NICE guidelines. 2014.

[9] Fransen M, McConnell S, Harmer AR, Van der Esch M, Simic M, Bennell KL. Exercise for osteoarthritis of the knee. Cochrane Database Syst Rev. 2015;1. 10.1002/14651858.CD004376.pub3. 10.1002/14651858.CD004376.pub3 PMC10094004 25569281

[10] Goh SL, Persson MSM, Stocks J, Hou Y, Lin J, Hall MC, Doherty M, Zhang W (2019). Efficacy and potential determinants of exercise therapy in knee and hip osteoarthritis: a systematic review and meta-analysis. Ann Phys Rehabil Med.

[11] Hurley MV, Walsh NE, Mitchell HL, Pimm TJ, Patel A, Williamson E, Jones RH, Dieppe PA, Reeves BC (2007). Clinical effectiveness of a rehabilitation program integrating exercise, self-management, and active coping strategies for chronic knee pain: a cluster randomized trial. Arthritis Rheum.

[12] Garber CE, Blissmer B, Deschenes MR, Franklin BA, Lamonte MJ, Lee IM, Nieman DC, Swain DP (2011). American College of Sports Medicine position stand. Quantity and quality of exercise for developing and maintaining cardiorespiratory, musculoskeletal, and neuromotor fitness in apparently healthy adults: guidance for prescribing exercise. Med Sci Sports Exerc.

[13] Angst F, Aeschlimann A, Stucki G (2001). Smallest detectable and minimal clinically important differences of rehabilitation intervention with their implications for required sample sizes using WOMAC and SF-36 quality of life measurement instruments in patients with osteoarthritis of the lower extremities. Arthritis Rheum.

[14] Bennell KL, Dobson F, Hinman RS (2014). Exercise in osteoarthritis: moving from prescription to adherence. Best Pract Res Clin Rheumatol.

[15] Lyytinen T, Liikavainio T, Bragge T, Hakkarainen M, Karjalainen PA, Arokoski JPA (2010). Postural control and thigh muscle activity in men with knee osteoarthritis. J Electromyogr Kinesiol.

[16] Childs JD, Sparto PJ, Fitzgerald GK, Bizzini M, Irrgang JJ (2004). Alterations in lower extremity movement and muscle activation patterns in individuals with knee osteoarthritis. Clin Biomech (Bristol, Avon).

[17] Hubley-Kozey CL, Deluzio KJ, Landry SC, McNutt JS, Stanish WD (2006). Neuromuscular alterations during walking in persons with moderate knee osteoarthritis. J Electromyogr Kinesiol.

[18] Hortobagyi T, Westerkamp L, Beam S, Moody J, Garry J, Holbert D, DeVita P (2005). Altered hamstring-quadriceps muscle balance in patients with knee osteoarthritis. Clin Biomech.

[19] Winby CR, Gerus P, Kirk TB, Lloyd DG (2013). Correlation between EMG-based co-activation measures and medial and lateral compartment loads of the knee during gait. Clin Biomech.

[20] Heiden TL, Lloyd DG, Ackland TR (2009). Knee joint kinematics, kinetics and muscle co-contraction in knee osteoarthritis patient gait. Clin Biomech.

[21] Brandon SCE, Miller RH, Thelen DG, Deluzio KJ (2014). Selective lateral muscle activation in moderate medial knee osteoarthritis subjects does not unload medial knee condyle. J Biomech.

[22] Hodges PW, van den Hoorn W, Wrigley TV, Hinman RS, Bowles K-A, Cicuttini F, Wang Y, Bennell K (2016). Increased duration of co-contraction of medial knee muscles is associated with greater progression of knee osteoarthritis. Man Ther.

[23] Hatfield GL, Costello KE, Astephen Wilson JL, Stanish WD, Hubley-Kozey CL (2020). Baseline gait muscle activation patterns differ for osteoarthritis patients who undergo total knee arthroplasty 5–8 years later from those who do not. Arthritis Care Res.

[24] Preece SJ, Jones RK, Brown CA, Cacciatore TW, Jones AK (2016). Reductions in co-contraction following neuromuscular re-education in people with knee osteoarthritis. BMC Musculoskelet Disord.

[25] Odole A, Ekediegwu E, Ekechukwu END, Uchenwoke C (2019). Correlates and predictors of pain intensity and physical function among individuals with chronic knee osteoarthritis in Nigeria. Musculoskelet Sci Pract.

[26] Sharma A, Kudesia P, Shi Q, Gandhi R (2016). Anxiety and depression in patients with osteoarthritis: impact and management challenges. Open Access Rheumatol.

[27] Scopaz KA, Piva SR, Wisniewski S, Fitzgerald GK (2009). Relationships of fear, anxiety, and depression with physical function in patients with knee osteoarthritis. Arch Phys Med Rehabil.

[28] Silva Guerrero AV, Maujean A, Campbell L, Sterling M (2018). A systematic review and meta-analysis of the effectiveness of psychological interventions delivered by physiotherapists on pain, disability and psychological outcomes in musculoskeletal pain conditions. Clin J Pain.

[29] Denneny D, Frijdal A, Bianchi-Berthouze N, Greenwood J, McLoughlin R, Petersen K, Singh A, Williams ACC. The application of psychologically informed practice: observations of experienced physiotherapists working with people with chronic pain. Physiotherapy. 2020;106:163–73. 10.1016/j.physio.2019.01.014. 10.1016/j.physio.2019.01.014 30930053

[30] O'Sullivan PB, Caneiro JP, O'Keeffe M, Smith A, Dankaerts W, Fersum K, O'Sullivan K (2018). Cognitive functional therapy: an integrated behavioral approach for the targeted Management of Disabling low Back Pain. Phys Ther.

[31] Koes BW, van Tulder M, Lin C-WC, Macedo LG, McAuley J, Maher C (2010). An updated overview of clinical guidelines for the management of non-specific low back pain in primary care. Eur Spine J.

[32] Veenhof C, Köke AJ, Dekker J, Oostendorp RA, Bijlsma JW, van Tulder MW, van den Ende CH (2006). Effectiveness of behavioral graded activity in patients with osteoarthritis of the hip and/or knee: a randomized clinical trial. Arthritis Rheum.

[33] Hurley M, Dickson K, Hallett R, Grant R, Hauari H, Walsh N, et al. Exercise interventions and patient beliefs for people with hip, knee or hip and knee osteoarthritis: amixedmethods review. Cochrane Database Syst Rev. 2018;4. 10.1002/14651858.CD004376.pub3. 10.1002/14651858.CD010842.pub2 PMC6494515 29664187

[34] Duncan E, O'Cathain A, Rousseau N, Croot L, Sworn K, Turner KM, Yardley L, Hoddinott P (2020). Guidance for reporting intervention development studies in health research (GUIDED): an evidence-based consensus study. BMJ Open.

[35] Altman R, Alarcon G, Appelroth D (1986). The American College of Rheumatology criteria for the classification and reporting of osteoarthritis of the knee. Arthritis Rheum.

[36] O'Cathain A, Croot L, Duncan E, Rousseau N, Sworn K, Turner KM, Yardley L, Hoddinott P (2019). Guidance on how to develop complex interventions to improve health and healthcare. BMJ Open.

[37] Bartholomew LK, Parcel GS, Kok G, Gottlieb NH, Fernández ME (2011). Planning health promotion programs: an intervention mapping approach.

[38] Michie S, van Stralen MM, West R (2011). The behaviour change wheel: a new method for characterising and designing behaviour change interventions. Implement Sci.

[39] Behaviour change: individual approaches: Public health guideline [PH49]: NICE guidelines. 2014.

[40] Sekhon M, Cartwright M, Francis JJ (2017). Acceptability of healthcare interventions: an overview of reviews and development of a theoretical framework. BMC Health Serv Res.

[41] Braun V, Clarke V (2006). Using thematic analysis in psychology. Qual Res Psychol.

[42] Turcot K, Sagawa Y, Hoffmeyer P, Suvà D, Armand S (2015). Multi-joint postural behavior in patients with knee osteoarthritis. Knee.

[43] Yasuda T, Togawa D, Hasegawa T, Yamato Y, Kobayashi S, Yoshida G, Banno T, Arima H, Oe S, Hoshino H, Koyama H, Hanada M, Imada T, Matsuyama Y (2020). Relationship between knee osteoarthritis and Spinopelvic sagittal alignment in volunteers over 50 years of age. Asian Spine J.

[44] Wang WJ, Liu F, Zhu YW, Sun MH, Qiu Y, Weng WJ (2016). Sagittal alignment of the spine-pelvis-lower extremity axis in patients with severe knee osteoarthritis: a radiographic study. Bone Joint Res.

[45] Tauchi R, Imagama S, Muramoto A, Tsuboi M, Ishiguro N, Hasegawa Y (2015). Influence of spinal imbalance on knee osteoarthritis in community-living elderly adults. Nagoya J Med Sci.

[46] Janda V, Jull G. Muscles and motor control in low back pain: assessment and management. Physical Therapy of the Low Back. T Twomey, Churchill livingstone New York. 1987:p253–78.

[47] Kendall F, McCreary E, Provance P, Rodgers M, Romani W: Testing and function with posture and pain: Lippincoll Williams & Wilkins; 2005.

[48] Preece SJ, Alghamdi W (2020). Inter-individual variation in hip flexor length may explain differences in trunk flexion during walking in people with knee osteoarthritis. Osteoarthr Cartil.

[49] Preece SJ, Tan YF, Alghamdi TD, Frances A: Comparison of pelvic tilt before and after hip flexor stretching in healthy adults Journal of Manipulative and Physiological Therapeutics (accepted) 2020. 10.1016/j.jmpt.2020.09.006 34090549

[50] Preece SJ, Alghamdi W (2021). The effect of increasing trunk flexion during normal walking. Gait & Posture.

[51] Alghamdi W, Preece SJ (2020). How does normal variability in trunk flexion affect lower limb muscle activity during walking?. Hum Mov Sci.

[52] Preece SJ, Algarni AS, Jones RK (2019). Trunk flexion during walking in people with knee osteoarthritis. Gait Posture.

[53] Preece SJ, Alghamdi W, Jones R (2019). Could increased trunk flexion underlie alterations in knee muscle activity in people with knee OA?. Osteoarthr Cartil.

[54] Csapo R, Maganaris CN, Seynnes OR, Narici MV (2010). On muscle, tendon and high heels. J Exp Biol.

[55] Wisdom KM, Delp SL, Kuhl E (2015). Use it or lose it: multiscale skeletal muscle adaptation to mechanical stimuli. Biomech Model Mechanobiol.

[56] Boukabache A, Preece SJ, Brookes N (2021). Prolonged sitting and physical inactivity are associated with limited hip extension: a cross-sectional study. Musculoskelet Sci Pract.

[57] Heneghan NR, Baker G, Thomas K, Falla D, Rushton A (2018). What is the effect of prolonged sitting and physical activity on thoracic spine mobility? An observational study of young adults in a UK university setting. BMJ Open.

[58] Mier A, Brophy C, Estenne M, Moxham J, Green M, De Troyer A (1985). Action of abdominal muscles on rib cage in humans. J Appl Physiol (Bethesda, Md : 1985).

[59] Lluch E, Torres R, Nijs J, Van Oosterwijck J (2014). Evidence for central sensitization in patients with osteoarthritis pain: a systematic literature review. Eur J Pain (London, England).

[60] Finan PH, Buenaver LF, Bounds SC, Hussain S, Park RJ, Haque UJ, Campbell CM, Haythornthwaite JA, Edwards RR, Smith MT (2013). Discordance between pain and radiographic severity in knee osteoarthritis: findings from quantitative sensory testing of central sensitization. Arthritis Rheum.

[61] Girbes EL, Nijs J, Torres-Cueco R, Cubas CL (2013). Pain treatment for patients with osteoarthritis and central sensitization. Phys Ther.

[62] Martindale JC, Wilson AW, Reeve AJ, Chessell IP, Headley PM (2007). Chronic secondary hypersensitivity of dorsal horn neurones following inflammation of the knee joint. Pain.

[63] Lumley MA, Cohen JL, Borszcz GS, Cano A, Radcliffe AM, Porter LS, Schubiner H, Keefe FJ (2011). Pain and emotion: a biopsychosocial review of recent research. J Clin Psychol.

[64] Mason KJ, O'Neill TW, Lunt M, Jones AKP, McBeth J (2018). Psychosocial factors partially mediate the relationship between mechanical hyperalgesia and self-reported pain. Scand J Pain.

[65] Lopez-Bravo MD, Zamarron-Cassinello MD, Touche RL, Munoz-Plata R, Cuenca-Martinez F, Ramos-Toro M. Psychological factors associated with functional disability in patients with hip and knee osteoarthritis. Behav Med. 2020:1–11. 10.1080/08964289.2020.1813682. 10.1080/08964289.2020.1813682 32910744

[66] Creamer P, Lethbridge-Cejku M, Hochberg MC (1999). Determinants of pain severity in knee osteoarthritis: effect of demographic and psychosocial variables using 3 pain measures. J Rheumatol.

[67] Degerstedt Å, Alinaghizadeh H, Thorstensson CA, Olsson CB (2020). High self-efficacy – a predictor of reduced pain and higher levels of physical activity among patients with osteoarthritis: an observational study. BMC Musculoskelet Disord.

[68] Hodges PW, Tucker K (2011). Moving differently in pain: a new theory to explain the adaptation to pain. Pain.

[69] Hodges PW, Tsao H, Sims K (2015). Gain of postural responses increases in response to real and anticipated pain. Exp Brain Res.

[70] Butera KA, Fox EJ, George SZ (2016). Toward a transformed understanding: from pain and movement to pain with movement. Phys Ther.

[71] Lewek MD, Ramsey DK, Snyder-Mackler L, Rudolph KS (2005). Knee stabilization in patients with medial compartment knee osteoarthritis. Arthritis Rheum.

[72] Sritharan P, Lin YC, Richardson SE, Crossley KM, Birmingham TB, Pandy MG (2016). Musculoskeletal loading in the symptomatic and asymptomatic knees of middle-aged osteoarthritis patients. J Orthop Res.

[73] Geisser ME, Haig AJ, Wallbom AS, Wiggert EA (2004). Pain-related fear, lumbar flexion, and dynamic EMG among persons with chronic musculoskeletal low back pain. Clin J Pain.

[74] Pakzad M, Fung J, Preuss R (2016). Pain catastrophizing and trunk muscle activation during walking in patients with chronic low back pain. Gait Posture.

[75] Stefanik JJ, Frey-Law L, Segal NA, Niu J, Lewis CE, Nevitt MC, Neogi T (2020). The Relation of Peripheral and Central Sensitization to Muscle Co-contraction: The MOST Study. Osteoarthritis Cartil.

[76] Andriacchi TP, Mündermann A, Smith RL, Alexander EJ, Dyrby CO, Koo S (2004). A framework for the in vivo pathomechanics of osteoarthritis at the knee. Ann Biomed Eng.

[77] Andriacchi TP, Mundermann A (2006). The role of ambulatory mechanics in the initiation and progression of knee osteoarthritis. Curr Opin Rheumatol.

[78] Giggins OM, Persson UM, Caulfield B (2013). Biofeedback in rehabilitation. J Neuroeng Rehabil.

[79] Kok G, Gottlieb NH, Peters GJ, Mullen PD, Parcel GS, Ruiter RA, Fernández ME, Markham C, Bartholomew LK (2016). A taxonomy of behaviour change methods: an intervention mapping approach. Health Psychol Rev.

[80] Louw A, Diener I, Butler DS, Puentedura EJ (2011). The effect of neuroscience education on pain, disability, anxiety, and stress in chronic musculoskeletal pain. Arch Phys Med Rehabil.

[81] De Troyer A (1983). Mechanical role of the abdominal muscles in relation to posture. Respir Physiol.

[82] Ivanenko Y, Gurfinkel VS (2018). Human Postural Control. Front Neurosci.

[83] Jeannerod M (1995). Mental imagery in the motor context. Neuropsychologia.

[84] Rutherford DJ, Hubley-Kozey CL, Stanish WD, Dunbar MJ (2011). Neuromuscular alterations exist with knee osteoarthritis presence and severity despite walking velocity similarities. Clin Biomech.

[85] Huang H, Wolf SL, He J (2006). Recent developments in biofeedback for neuromotor rehabilitation. J Neuroeng Rehabil.

[86] van der Esch M, Holla JF, van der Leeden M, Knol DL, Lems WF, Roorda LD, Dekker J (2014). Decrease of muscle strength is associated with increase of activity limitations in early knee osteoarthritis: 3-year results from the cohort hip and cohort knee study. Arch Phys Med Rehabil.

[87] Holla JFM, Sanchez-Ramirez DC, Van Der Leeden M, Ket JCF, Roorda LD, Lems WF, Steultjens MPM, Dekker J (2014). The avoidance model in knee and hip osteoarthritis: a systematic review of the evidence. J Behav Med.

[88] Beckwée D, Vaes P, Cnudde M, Swinnen E, Bautmans I (2013). Osteoarthritis of the knee: why does exercise work? A qualitative study of the literature. Ageing Res Rev.

[89] Richards RE, van den Noort JC, van der Esch M, Booij MJ, Harlaar J (2018). Effect of real-time biofeedback on peak knee adduction moment in patients with medial knee osteoarthritis: Is direct feedback effective?. Clin Biomech (Bristol, Avon).

[90] Booij MJ, Richards R, Harlaar J, van den Noort JC (2020). Effect of walking with a modified gait on activation patterns of the knee spanning muscles in people with medial knee osteoarthritis. Knee.

[91] O'Neill TW, Felson DT (2018). Mechanisms of osteoarthritis (OA) pain. Curr Osteop Rep.

[92] Anandacoomarasamy A, Caterson I, Sambrook P, Fransen M, March L (2008). The impact of obesity on the musculoskeletal system. Int J Obes (2005).

[93] Gerhart JI, Burns JW, Post KM, Smith DA, Porter LS, Burgess HJ, Schuster E, Buvanendran A, Fras AM, Keefe FJ (2017). Relationships between sleep quality and pain-related factors for people with chronic low Back pain: tests of reciprocal and time of day effects. Ann Behav Med.

[94] McCracken LM (2005). Social context and acceptance of chronic pain: the role of solicitous and punishing responses. Pain.

[95] Suri P, Morgenroth DC, Kwoh CK, Bean JF, Kalichman L, Hunter DJ (2010). Low Back pain and other musculoskeletal pain comorbidities in individuals with symptomatic osteoarthritis of the knee: data from the osteoarthritis initiative. Arthritis Care Res.

