# Correction to: A new integrated behavioural intervention for knee osteoarthritis: development and pilot study

**DOI:** 10.1186/s12891-021-04993-0

**Published:** 2022-01-25

**Authors:** Stephen J. Preece, Nathan Brookes, Anita E. Williams, Richard K. Jones, Chelsea Starbuck, Anthony Jones, Nicola E. Walsh

**Affiliations:** 1 Centre for Health Sciences Research, University of Salford, Manchester, M6 6PU UK; 2 Physiotherapy Department, Salford Royal NHS Foundation Trust, Salford, M6 8HD UK; 3 Human Pain Research Group, University of Manchester, Clinical Sciences Building, Salford Royal NHS Foundation Trust, Salford, M6 8HD UK; 4 Faculty of Health and Applied Sciences, University of the West of England, Bristol, BS16 1DD UK

**Correction to: BMC Musculoskelet Disord 22, 526 (2021)**

**https://doi.org/10.1186/s12891-021-04389-0**

Following the publication of the original article [1] the authors noticed a typo error in Table 4. The data “5 (2.1)” should be changed to “2.5 (2.1)”.

The original article [1] has been updated.
