# Supplementary material for: A new integrated behavioural intervention for knee osteoarthritis: development and pilot study
Source: BMC Musculoskelet Disord. 2021 Jun 8;22:526. doi: 10.1186/s12891-021-04389-0 (PMC8188786; doi:10.1186/s12891-021-04389-0)
Supplement: Supplementary file 1 — Additional file 1. Full description of the final intervention. [file 12891_2021_4389_MOESM1_ESM.docx]

A new integrated behavioural intervention for knee osteoarthritis: development and pilot study

# Appendix 1: Full description of the final intervention

The final intervention consisted of five components. We have provided a detailed explanation of each component, explaining how it was tailored to an individual patient’s needs.

***Component 1: Making sense of pain***

We used persuasive communication and imagery (through animated videos) to challenge the belief that knee OA pain is the inevitable result of “wear and tear” and to convey the idea that increased muscle activation will increase knee loads, potentially exacerbating pain. See [www.cogmustherapy.com/BMC_example_2](http://www.cogmustherapy.com/BMC_example_2) for an example. We also created animated videos to explain that “tensing muscles” in response to pain could further exacerbate pain. Drawing on the proven efficacy of neuroscience education [1], we created educational materials [2] to explain the idea that brain processing will modulate the pain experience; that pain is not always a true representation of the status of tissues; and that psychosocial factors can shape the pain experience [1]. We emphasised the idea of “escaping” from vicious cycles, both in terms of motor responses to pain and central sensitisation (Figure 5), and the need to raise consciousness of habitual responses to pain. This component was tailored by increasing/decreasing the focus on central sensitisation which was determined by applying established principles for the recognition of central sensitisation in musculoskeletal pain [3].

***Component 2: General relaxation***

There were two primary aims to teaching general relaxation, firstly to initiate the process of postural deconstruction (see below) and secondly to raise consciousness of both emotional and muscular responses to pain. This focus is consistent with previous research supporting the use of relaxation interventions for arthritis pain [4] and the use of cognitive behavioural therapy interventions which incorporate relaxation for knee OA [5]. We targeted three easy-to-observe characteristics of relaxation. The first was active contraction of the quadriceps muscles which was monitored using either EMG biofeedback or a patella glide test. Secondly, we monitored the degree of resistance to passive limb movement when the patient was lying supine. Finally, we monitored low level contraction of the abdominal muscles (focusing primarily on rectus abdominus) using manual palpation, along with a modified version of the Hi-Lo breathing assessment [6]. For the breathing assessment, reduced movement of the abdomen was taken as secondary confirmation of increased abdominal muscle tone, which has been associated with reduced abdominal volume during respiration [7].

The abdominal assessment was carried out in supine lying and in standing. Interestingly, all 21 participants demonstrated elevated abdominal tone in standing along with a tendency for scapula elevation when instructed to take a deep breath. To communicate the idea that increased abdominal tone would alter breathing patterns, we created an animated video that can be accessed at [www.cogmustherapy.com/BMC_example_3](http://www.cogmustherapy.com/BMC_example_3). To train relaxation of the abdominal muscles, patients were provided with a set of instructional videos which contrasted the altered breathing pattern with the target pattern (full diaphragmatic movement with minimal shoulder motion). These videos explained the mechanics of breathing and guided the development of a diaphragmatic breathing pattern, first in lying and then in standing. To reduce resistance to passive limb movement, the physiotherapist worked with the patient in a supine position to facilitate awareness of muscular holding. Using EMG biofeedback (or patella glide), patients were taught to develop a sense of relaxing-contracting the quadriceps, first in supine/sitting and then in a standing position. This intervention component was tailored by using customised breathing retraining and by the degree of focus on the teaching of the release of resistance to passive limb movements.

***Component 3: Postural deconstruction***

This intervention component focused on improving the organisation of postural muscle tone. Whereas postural alignment describes the relative orientation of body segments, postural tone can be thought of as “tonic (sustained) activation of muscles in order to provide specific postural attitude and generate force against the ground to keep the limbs extended” [8]. Based on the framework in Figure 4, we suggest that passive stiffness of the hip/trunk muscles will drive a re-organisation of postural tone, through a set of compensatory increases in tonic muscle activity required to maintain upright standing and establish normal gaze direction. In line with this idea, the task for the physiotherapist is to unpick (deconstruct) patterns of postural tone, facilitate the resetting of proprioceptive relationships between body segments and to re-establish a more optimal balance of postural muscle activity.

We devised a clinical protocol incorporating manual muscle testing, palpation and visual assessment along with EMG assessment of knee muscle activity. This enabled the physiotherapist to identify hip/trunk stiffness and interconnected patterns of compensatory postural tone. Following this assessment, animated instructional videos were used to provide the patient with a conceptual understanding of their individual muscle/postural patterns. Patients were then guided through a set of procedures, the first of which required them to move from a flexed to an upright position. Performed slowly, this allowed the physiotherapist to identify the “tension point” at which there was a marked increase in tonic muscle activity in order to overcome hip flexor or abdominal stiffness. At this point, there was often an observable increase in quadriceps EMG which was visualised using the biofeedback software. Patients were then guided through a range of procedures, using diaphragmatic breathing, segmental dissociation, positional awareness and gentle stretching to both reduce hip/trunk muscle stiffness and raise awareness of compensatory postural tone. Following these procedures, the aim was to achieve an upright standing position without triggering compensatory tone.

This intervention component was highly tailored and based on an individualised assessment, the use of individually selected videos and an individually developed retraining plan. To illustrate the idea of postural deconstruction, Figure 2 shows how compensatory tone can be triggered in the knee extensors, secondary to passive stiffness of the hip flexors. In Figure 2a, a flexed position is adopted, and the hip flexor is in a slack position. Figure 2b shows the tension point at which there is no slack in the hip flexor, but an upright position has not been achieved. Figure 2c show compensatory knee flexion required to facilitate posterior pelvic rotation and upright standing. This knee flexion will trigger activation of the knee extensor muscles (compensatory tone). In this scenario, retraining would involve a focus on positional awareness of the knee, hip flexor lengthening/relaxation and a focus on moving past the tension point without triggering compensatory knee muscle contraction (visualised using EMG biofeedback). In addition, the physiotherapist would focus on compensatory tone in the spinal erector muscles, which may be triggered to increase lumbar extension in order to achieve an upright position. EMG activity in the hamstring muscles would also be monitored as this may increase to balance any anterior shift in the centre of mass.

With our theoretical framework (Figure 4), increased passive stiffness of the hip/trunk muscles is associated with physical inactivity and prolonged sitting [9, 10]. This concept was explained to patients and they were encouraged to take regular walking exercise and break up periods of prolonged sitting by walking or standing for a few minutes. As part of this component, the physiotherapist challenged any beliefs relating to exercise avoidance. Patients were also encouraged to observe how postural tone in sitting, such as low-level hip flexor activity [11], might be carried through into standing, especially after sitting for prolonged periods. In addition, patients were encouraged to use the enhanced body awareness they had developed during the process of postural deconstruction to “make sense of stiffness.” Specifically, patients were encouraged to recognise the difference between intrinsic joint stiffness (resulting from limitations in the joint and capsular structures) and muscle stiffness, which may result from elevated postural tone.

It is important to contrast the clinical procedures developed for this intervention component with muscle imbalance physiotherapy [12, 13]. Central to muscle imbalance physiotherapy is the idea of an imbalance between the strength and/or flexibility of the agonist or antagonist muscle acting over a joint [13]. The task for the clinician is to prescribe specific stretching and strengthening exercises to correct this imbalance. In contrast, our approach is based on the idea of postural abnormality being the result of increased stiffness of muscles which attach directly onto the pelvis and the associated biomechanical compensations required to stand upright. We highlight that our approach has no focus on muscle strengthening. Instead, it teaches patients to make subtle changes to postural muscle activity through body-awareness training.

***Component 4: Responding differently to pain***

Building on component 1 (making sense of pain), this component made use of EMG biofeedback to identify the presence of knee muscle contraction which was related to pain, pain expectations or pain-related beliefs. Using animated videos, we reiterated the idea that inappropriate muscle contraction could perpetuate the pain experience. EMG biofeedback was then used to raise consciousness of knee muscle activity in standing and to explore how expectations from pain provoking activities could trigger excessive muscular contraction. For example, many patients found stepping down painful and would over activate their knee muscles in anticipation of pain from this movement. Using EMG biofeedback, patients could observe this behaviour and were taught to ‘downregulate’ muscle activity before such tasks. In addition, we recognised that localised changes in knee muscle activity, related to previous pain experience, may trigger compensatory postural tone. Therefore, learning from this intervention component was integrated into the postural deconstruction procedures, described above.

We suggest that this approach, of teaching patients to observe and consciously influence their muscular responses to anticipated pain, has parallels with cognitive restructuring techniques which have been used previously in CBT programmes [14] for knee OA [15]. Specifically, the increased muscle activation is seen as a maladaptive pattern (problem behaviour), which can be brought into conscious awareness so that it can be substituted for a healthy behaviour (more relaxed knee muscle) through counter conditioning. Such muscle patterns are likely to be connected to past experience and beliefs about pain. Therefore, the physiotherapist used this opportunity to continue to challenge patient’s beliefs around the chronic nature of knee OA pain and also challenge the idea that certain movements should be avoided. Through this process, patients were continually encouraged to reflect on their fear/anxiety about pain and to develop a sense of how such responses could be linked to maladaptive muscular behaviour. This component of the intervention was tailored by allowing the patient to choose the pain provoking movements and the degree to which the emotional beliefs underlying these patterns were explored during clinical sessions.

***Component 5: Functional muscle retraining***

This final component was carried out once patients had developed competence in postural deconstruction and were able to minimise maladaptive muscular responses to pain. The first stage of this training involved the use of EMG biofeedback to enable patients to develop the ability to momentarily balance on one leg with minimal activation of knee muscles of the stance limb, as if about to initiate a step forwards/up/down. Once this had been mastered, the focus was on a set of patient-selected functional tasks (walking, stepping down, stepping up, sit-to-stand or stand-to-sit). For this second stage, the patient’s EMG muscle profile was initially visualised against a healthy template, created from an EMG database of signals collected from a cohort of 45 healthy volunteers (aged 18-83). This visualisation provided the patient with a conceptual understanding of how their muscle patterns differed from those of a person without knee pain.

With a conceptual understanding of their knee muscle pattern, patients were instructed to repeat the selected task and to mentally experiment with a specific motor command. The precise choice of motor command was designed to normalise the muscle pattern and therefore selected based on the timing/magnitude of the patient’s EMG signal. In some cases, animated videos were used to help convey the desired motor commands, using motor imagery [16]. For example, many participants exhibited prolonged quadriceps activity into midstance of walking. By using an instruction such as “imagine a rope pulling the leg forwards as you walk”, the patient learned to associate the specific motor command with the desired motor behaviour, i.e. match their muscle pattern with the healthy template in the biofeedback software. In addition to using motor commands aimed at encouraging smooth movement, kinematic instruction was also employed where appropriate. For example, patients were discouraged from positioning their feet too anteriorly when performing a sit-to-stand movement as this requires greater acceleration of the centre of mass on movement initiation [17]. The functional muscle retraining was highly tailored as it used knowledge of patient’s muscle activation (EMG signals) to create an individual training programme.

# Figures


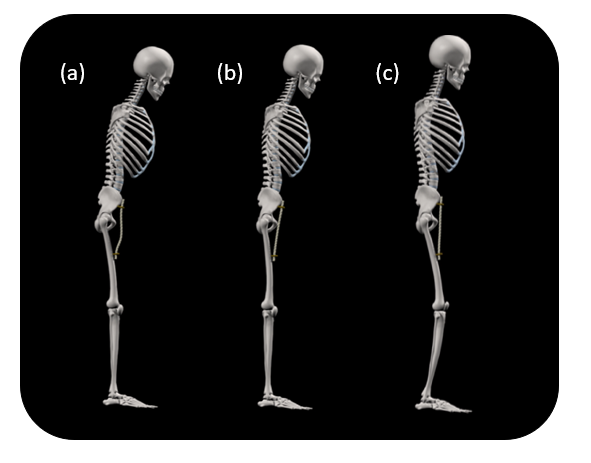


***Figure 2: (a, b) A passively stiff hip flexor (illustrated as a rope) prevents the pelvis returning to a neutral position in standing. (c) Biomechanical compensation for a passively stiff hip flexor, consisting of a flexed hip, knee and ankle and an increased lumbar lordosis. Note there is still a slight flexion of the trunk. A full animation of this pattern can be viewed at:*** [***www.cogmustherapy.com/BMC_example_1***](http://www.cogmustherapy.com/BMC_example_1).


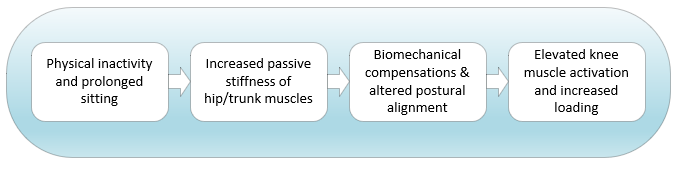


***Figure 4: Postural framework to explain elevated knee muscle activation
from increased passive stiffness of hip/trunk muscles***


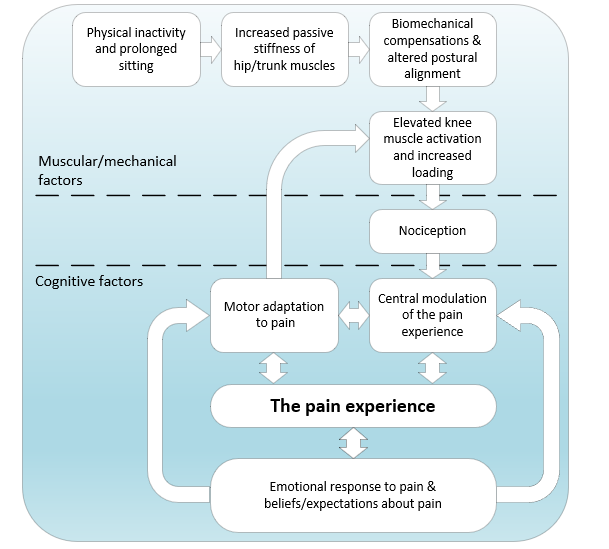


***Figure 5: Integrated behavioural framework***

# References

1. Louw A, Diener I, Butler DS, Puentedura EJ: **The effect of neuroscience education on pain, disability, anxiety, and stress in chronic musculoskeletal pain**. *Arch Phys Med Rehabil* 2011, **92**(12):2041-2056.

2. Nijs J, Paul van Wilgen C, Van Oosterwijck J, van Ittersum M, Meeus M: **How to explain central sensitization to patients with 'unexplained' chronic musculoskeletal pain: practice guidelines**. *Man Ther* 2011, **16**(5):413-418.

3. Nijs J, Van Houdenhove B, Oostendorp RA: **Recognition of central sensitization in patients with musculoskeletal pain: Application of pain neurophysiology in manual therapy practice**. *Man Ther* 2010, **15**(2):135-141.

4. Kwekkeboom KL, Gretarsdottir E: **Systematic review of relaxation interventions for pain**. *Journal of nursing scholarship : an official publication of Sigma Theta Tau International Honor Society of Nursing* 2006, **38**(3):269-277.

5. Murphy SL, Janevic MR, Lee P, Williams DA: **Occupational Therapist-Delivered Cognitive-Behavioral Therapy for Knee Osteoarthritis: A Randomized Pilot Study**. *American Journal of Occupational Therapy* 2018, **72**(5).

6. Courtney R, Cohen M, Reece J: **Comparison of the Manual Assessment of Respiratory Motion (MARM) and the Hi Lo Breathing Assessment in determining a simulated breathing pattern**. *International Journal of Osteopathic Medicine* 2009, **12**(3):86-91.

7. De Troyer A: **Mechanical role of the abdominal muscles in relation to posture**. *Respiration Physiology* 1983, **53**(3):341-353.

8. Ivanenko Y, Gurfinkel VS: **Human Postural Control**. *Frontiers in Neuroscience* 2018, **12**(171).

9. Boukabache A, Preece SJ, Brookes N: **Prolonged sitting and physical inactivity are associated with limited hip extension: A cross-sectional study**. *Musculoskeletal Science and Practice* 2021, **51**:102282.

10. Heneghan NR, Baker G, Thomas K, Falla D, Rushton A: **What is the effect of prolonged sitting and physical activity on thoracic spine mobility? An observational study of young adults in a UK university setting**. *BMJ Open* 2018, **8**(5):e019371.

11. Park RJ, Tsao H, Claus A, Cresswell AG, Hodges PW: **Changes in Regional Activity of the Psoas Major and Quadratus Lumborum With Voluntary Trunk and Hip Tasks and Different Spinal Curvatures in Sitting**. *Journal of Orthopaedic & Sports Physical Therapy* 2013, **43**(2):74-82.

12. Norris CM: **Spinal Stabilisation: 4. Muscle Imbalance and the Low Back**. *Physiotherapy* 1995, **81**(3):127-138.

13. Kendall F, McCreary E, Provance P, Rodgers M, Romani W: **Testing and function with Posture and Pain**: Lippincoll Williams & Wilkins; 2005.

14. Perini S, Titov N, Andrews G: **Clinician-assisted Internet-based treatment is effective for depression: randomized controlled trial**. *The Australian and New Zealand journal of psychiatry* 2009, **43**(6):571-578.

15. O'Moore KA, Newby JM, Andrews G, Hunter DJ, Bennell K, Smith J, Williams AD: **Internet Cognitive-Behavioral Therapy for Depression in Older Adults With Knee Osteoarthritis: A Randomized Controlled Trial**. *Arthritis Care & Research* 2018, **70**(1):61-70.

16. Jeannerod M: **Mental imagery in the motor context**. *Neuropsychologia* 1995, **33**(11):1419-1432.

17. Cacciatore TW, Mian OS, Peters A, Day BL: **Neuromechanical interference of posture on movement: evidence from Alexander technique teachers rising from a chair**. *Journal of Neurophysiology* 2014, **112**(3):719-729.
